# A concise and practical stereoselective synthesis of ipragliflozin L-proline

**DOI:** 10.3762/bjoc.13.105

**Published:** 2017-06-01

**Authors:** Shuai Ma, Zhenren Liu, Jing Pan, Shunli Zhang, Weicheng Zhou

**Affiliations:** 1 State Key Lab of New Drug & Pharmaceutical Process, Shanghai Key Lab of Anti-Infectives, Shanghai Institute of Pharmaceutical Industry, China State Institute of Pharmaceutical Industry, No. 285, Gebaini Rd., Shanghai 201203, P. R. of China

**Keywords:** arylzinc derivative, β-*C*-arylglucoside, diastereomer impurity, ipragliflozin L-proline, stereoselective synthesis

## Abstract

A concise and practical stereoselective synthesis of ipragliflozin L-proline was presented starting from 2-[(5-iodo-2-fluorophenyl)methyl]-1-benzothiophene and 2,3,4,6-tetra-*O*-pivaloyl-α-D-glucopyranosyl bromide without catalyst via iodine–lithium–zinc exchange. The overall yield was 52% in three steps and the product purity was excellent. Two key diastereomers were prepared with efficient and direct access to the α-*C*-arylglucoside.

## Introduction

**Figure 1:**
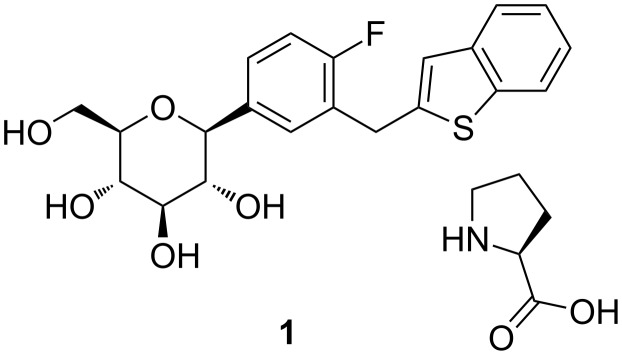
Structure of ipragliflozin L-proline.

Sodium-glucose co-transporter 2 (SGLT2) inhibitors are a new class of antidiabetes drugs, and dapagliflozin [1], canagliflozin [2], empagliflozin [3], ipragliflozin [4], tofogliflozin [5], luseogliflozin [6] have been approved for the treatment of Type 2 diabetes mellitus (T2DM). One of them, (ipragliflozin L-proline **1**, (1*S*)-1,5-anhydro-1-*C*-{3-[(1-benzothiophen-2-yl)methyl]-4-fluorophenyl}-D-glucitol (2*S*)-pyrrolidine-2-carboxylic acid (1:1), Figure 1), was launched into the Japanese market in January 2014 [7–8]. Due to its efficacy and safety, **1** can be used as monotherapy or in combination with other hypoglycemic agents.

Only a few methods have been found in the literature about the synthesis of **1**. It was reported that aryllithium reacted with per-hydroxy-protected D-glucono-1,5-lactone at −78 °C to obtain the lactol, followed by reduction with a large bulk silyl hydride in order to get high β-selectivity [9–10]. This approach included six steps such as addition, methyl etherification, acetylation, reduction, deprotection and cocrystallization with L-proline to get **1** with a total yield of 32.89%. The subsequent improvement reported included the replacement of aryllithium by aryl Grignard reagents, raising the reaction temperature up to −20 °C [11]. However, the yield was not obviously improved.

**Scheme 1:**
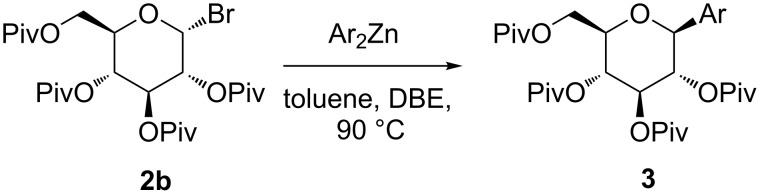
Stereoselective synthesis of *C*-aryl glycoside by Lemarie.

SGLT-2 inhibitors have the common pharmacophore of β-*C*-arylglucoside, and the synthesis of β-*C*-arylglucoside including the usage of arylzinc [12], arylalane [13], and anomeric stannane [14] were applied to prepare dapagliflozin and canagliflozin. However, these approaches might be difficult to scale up because of the involvements of complex synthetic procedures, chromatographic purification or expensive catalysts and ligands. Gong et al. [15] reported an approach to synthesise *C*-aryl glycosides based on a Negishi cross-coupling of arylzinc with protected glycosyl bromide in the presence of Ni-catalysts. An improved procedure with high stereoselectivity and in the absence of catalysts was reported subsequently by Lemarie et al. (Scheme 1) [12] to construct the anomeric chiral center of dapagliflozin and canagliflozin. But so far, it has not been found that this method was applied to synthesize **1**. In this paper, we report an efficient and practical method for the preparation of **1** through an arylzinc reagent.

## Results and Discussion

Herein, a concise and practical stereoselective synthesis of **1** with three steps was developed. The route initiated from compound **4a** and pivaloyl-protected glycosyl bromide **2b**, the β-*C*-arylglucoside **5** was obtained with high stereoselectivity in one step after a halogen–lithium exchange/transmetalation/coupling sequence. Cryogenic temperatures and catalysts were not required.

**Scheme 2:**
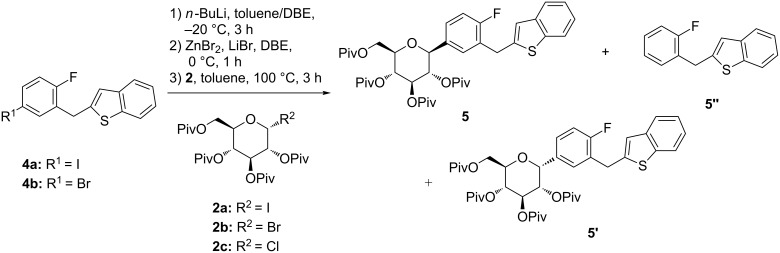
Stereoselective synthesis of β-*C*-arylglucoside **5**.

The key step was the diastereoselective synthesis of pivaloyl-protected **5** (Scheme 2). Thus, this reaction was studied in more detail to screen the best conditions, and the results were presented in Table 1.

**Table 1:** Conditions for the stereoselective synthesis of β-*C*-arylglucoside **5**.

Entry	Reactants	Zinc halide salt	Solvent	*T* (°C)	Crude product^a^ (**5:5’:5”**)	de^b^ (%)	Yield^c^ (%)

1	**4b**, **2b**	ZnBr_2_/LiBr (0.55 equiv)	Toluene/DBE^d^ (5:3)	−20	35%:0.5%:46%	97	48.4
2	**4a**, **2c**	ZnBr_2_/LiBr (0.55 equiv)	Toluene/DBE (5:3)	−20	50%:4%:36%	85	63.8
3	**4a**, **2a**	ZnI_2_/LiI (0.55 equiv)	Toluene/DBE (5:3)	−20	2%:0.3%:60%	74	−
4	**4a**, **2b**	ZnBr_2_/LiBr (0.55 equiv)	Toluene/DBE (5:3)	−20	68%:2%:21%	94	77.7
5	**4a**, **2b**	ZnBr_2_/LiBr (1.0 equiv)	Toluene/DBE (5:3)	−20	65%:2%:24%	94	75.6
6	**4a**, **2b**	ZnBr_2_/LiBr (0.55 equiv)	Toluene/DBE (5:3)	−10	34%:1%:46%	94	46.7
7	**4a**, **2b**	ZnBr_2_/LiBr(0.55 equiv)	Toluene/DBE (5:3)	−30	67%:2%:23%	94	76.5
8^e^	**4a**, **2b**	ZnBr_2_/LiBr (0.55 equiv)	Toluene/DBE (5:3)	−20	55%:2%:32%	93	78.9
9^f^	**4a**, **2b**	ZnBr_2_/LiBr (0.55 equiv)	Toluene/DBE (5:3)	−20	68%:2%:20%	94	77.9
10	**4a**, **2b**	ZnBr_2_/LiBr (0.55 equiv)	Toluene/THF (5:3)	−20	2%:0%:14%	0^g^	–
11	**4a**, **2b**	ZnBr_2_/LiBr (0.55 equiv)	Toluene/MTBE^h^ (5:3)	−20	5%:0%:67%	0^g^	6.5
12	**4a**, **2b**	ZnBr_2_/LiBr (0.55 equiv)	Toluene/CPME^i^ (5:3)	−20	33%:0.4%:49%	98	47
13^j^	**4a**, **2b**	ZnBr_2_/LiBr (0.55 equiv)	Toluene/DBE (5:3)	−20	66%:2%:22%	94	62.8^k^
14^l^	**4a**, **2b**	ZnBr_2_/LiBr (0.55 equiv)	Toluene/DBE (5:3)	−20	68%:2.5%:21%	93	65.3^k^

^a^Determined by HPLC at 263 nm, the content of **5’’** might be over-estimated since the different UV response between compound **5** and **5’’**. This may explain why the isolated yield after chromatography was higher than the content of **5** in the crude; ^b^diastereomeric excess of **5** and **5’**; ^c^isolated yield for **5** after column chromatography (entries 1–12); ^d^di-*n*-butyl ether; ^e^**4a** was added in 1.2 equiv; ^f^**2b** was added in 1.2 equiv; ^g^no diastereomer was detected; ^h^methyl *tert*-butyl ether; ^i^cyclopentyl methyl ether; ^j^20 g of **4a** was added; ^k^isolated by recrystallization; ^l^50 g of **4a** was added.

On the basis of literature [12], 2-[(5-bromo-2-fluorophenyl)methyl]-1-benzothiophene (**4b**) [9] was converted to the arylzinc species by the reaction with *n*-BuLi in the mixed solvent (toluene/DBE), followed by transmetalation with ZnBr/LiBr complex in DBE, and then the active species reacted with 2,3,4,6-tetra-*O*-pivaloyl-α-D-glucopyranosyl bromide (**2b**) [16]. But the reaction gave only 35% of desired **5** although it had a high diastereomeric excess (de) value (97%). The main byproduct was the debrominated compound **5”** determined by NMR and MS analysis (Table 1, entry 1). The reaction between 2-[(5-iodo-2-fluorophenyl)methyl]-1-benzothiophene (**4a**) [9] and pivaloyl-protected glycosyl halides **2** were further explored. The reaction of chloride **2c** [17] with **4a** provided a better result (Table 1, entry 2). With respect to the iodide **2a** [18], due to its thermal and chemical instability, there was only 2% of **5** detected, and the content of **5”** was up to 60% (Table 1, entry 3). Fortunately, the reaction of **4a** with 1.0 equiv **2b** gave a good result, the amount of **5”** was greatly reduced (Table 1, entry 4), while the content of **5** in the crude product was increased to 68% and the isolated yield was 77.7%. It was presumable that a iodine–lithium–zinc exchange and the transmetalation proceeded better than a bromide–lithium–zinc exchange and transmetalation to produce the arylzinc derivative in situ in this reaction. However, further increasing the amount of zinc bromide to 1.0 equiv did not improve the reaction (Table 1, entry 5) and elevation of the reaction temperature to −10 °C (Table 1, entry 6) worsened the reaction, resulting in more formation of **5’’**. Additionally, no significant improvement was achieved by further decreasing the temperature (Table 1, entry 7).

In entries 1–7 (Table 1), the feed ratio of two reagents (**4** and **2**) was set as 1:1. From the above data, it was seen that the reaction was acccompanied by the deiodination of **4a**, but to our surprise, neither elevation of the ratio to 1.2 of compound **4a** (Table 1, entry 8) nor **2b** (Table 1, entry 9) improved the reaction. When other solvents were tested (Table 1, entries 10–12), the results showed that the reaction in toluene/DBE mixed solvent afforded the best result. Interestingly, it was also found that the reaction of **2b** with **4a** gave a similar de (Table 1, entries 1, 4–9 and 12–14) in different conditions although the content of **5** was greatly varied. According to the methodology in entry 4, the reaction was scaled up (50 g **4a**) (Table 1, entry 14), and the crude product was purified by recrystallization from methanol, and the desired product in 65.3% yield with HPLC purity 99.79% was obtained; the diastereomer **5’** was not detected.

**Scheme 3:**
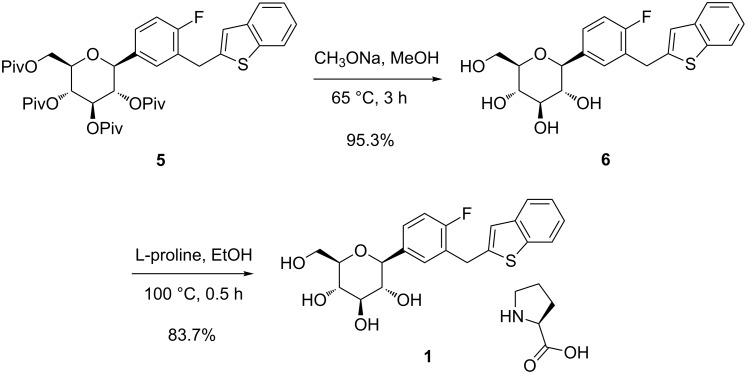
Synthesis of **1**.

The protecting groups of **5** were removed in presence of sodium methoxide in refluxing methanol, and then recrystallized to give ipragliflozin **6** in 95.3% yield. Compound **6** and L-proline was converted to the L-proline complex **1** as a cocrystal form in ethanol (Scheme 3). After filtration and drying, compound **1** was produced with 83.7% yield and 99.92% purity. The mp (dec) and optical rotation value of compound **1** was consistent with those in the literature reported [19], and the structure was confirmed by MS and NMR.

**Scheme 4:**
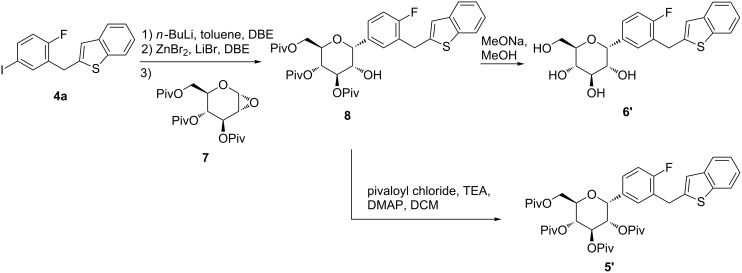
Synthesis of diastereomer **6’** and **5’**.

The α-anomers of **5** and **6** were the key impurities during the synthetic process. Pure compounds of **5’** and **6’** were prepared for the analytical references (Scheme 4). Compound **4a** was converted to the corresponding arylzinc derivative in situ, and the latter coupled with pivaloyl-protected 1,2-anhydroglucal **7** [20] to get the α-*C*-arylglucoside **8**. After removing the protecting groups in the presence of sodium methoxide, the α-anomer **6’** of ipragliflozin was obtained. The hydroxy group in 2-position of **8** was protected by pivaloyl chloride to generate **5’**. In the ^1^H NMR, the coupling constant of the anomeric proton for **6’** was 4.0 Hz, while the corresponding constant for **6** was 9.6 Hz, which was similar with those of canagliflozin [20].

## Conclusion

In conclusion, a concise and practical stereoselective synthesis of **1** starting from **4a** and **2b** was developed in a 52% overall yield with a purity of 99.92% over three steps. This method was easy to perform in large scale, and both the purity and yield were excellent. In addition, two key diastereomers were prepared with efficient and direct access to α-*C*-arylglucoside and they may be served as the references both in the monitoring the reaction and the quality control of the drug.

## Experimental

All solvents and reagents were of reagent grade and used without further purification. HPLC analyses were recorded using a Waters Arc HPLC System with 2998 PDA Detector or an Agilent 1260 HPLC System with DAD Detector. NMR spectra were recorded on a Bruker AV III 400 MHz spectrometer with TMS as an internal standard. Chemical shifts are quoted in parts per million (ppm). HRMS were recorded on a Waters Q-Tof Micro LCMS apparatus. Melting points were uncorrected and measured on a Büchi Melting Piont M-565 apparatus. Optical rotations were measured with an Autopol IV laboratory polarimeter.

**Preparation of (1*S*)-1,5-anhydro-1- {3-[(1-benzothiophen-2-yl)methyl]-4-fluorophenyl}-2,3,4,6-tetra-*O*-pivaloyl-D-glucitol (5):** A dry round-bottle flask was charged with solid ZnBr_2_ (16.8 g, 0.55 equiv), LiBr (6.5 g, 0.55 equiv) and anhydrous *n*-dibutyl ether (120 mL) under a nitrogen atmosphere. The resulting mixture was stirred at 50 °C until the solid dissolved, and then the mixture cooled back to room temperature and used as such for the next step. Compound **4a** (50 g, 135.8 mmol) was dissolved in anhydrous toluene (150 mL) and *n*-dibutyl ether (90 mL) under nitrogen atmosphere, and then the mixture was cooled to −20 °C, *n*-butyllithium in hexane 2.5 M (57.6 mL, 1.06 equiv) was added dropwise over 20 min. After beeing stirred for 3 h, a solution of ZnBr_2_–LiBr in *n*-dibutyl ether was added dropwise over 20 min at 0 °C. After stirring for 1 h, a solution of compound **2b** (78.7 g, 1.0 equiv) in toluene (120 mL) was added to the reaction mixture. The mixture was stirred at 100 °C for about 3 h until the reaction was completed (TLC detection). The mixture was quenched with 1 M HCl (300 mL), and then stirred for about 10 min and extracted. The organic phase was washed with water (300 mL), followed by concentration under reduced pressure to get a brown oil. The crude oil was crystallized from methanol to get a white solid 65.7 g. Yield 65.3%; purity (HPLC): 99.79%; mp 133–134.7 °C; [α]_D_^25^ = +14° (*c* 1, CHCl_3_); ^1^H NMR (400 MHz, CDCl_3_) δ 7.72 (d, *J* = 8.0 Hz, 1H), 7.64 (d, *J* = 8.4 Hz, 1H), 7.31–7.22 (m, 4H), 7.04 (t, *J* = 9.2 Hz, 1H), 6.97 (s, 1H), 5.40 (t, *J* = 9.6 Hz, 1H), 5.33–5.23 (m, 2H), 4.36 (d, *J* = 10.0 Hz, 1H), 4.25–4.16 (m, 3H), 4.10 (dd, *J* = 12.4 Hz, 4.0 Hz, 1H), 3.84–3.80 (m, 1H), 1.19 (s, 9H), 1.16 (s, 9H), 1.11 (s, 9H), 0.84 (s, 9H); ^13^C NMR (100 MHz, CDCl_3_) δ 178.1, 177.3, 176.4, 162.2, 159.8, 142.9, 139.9, 132.4, 130.7, 128.3, 126.6, 124.2, 123.8, 123.0, 122.0, 115.6, 80.3, 73.6, 72.0, 67.9, 61.7, 38.7, 30.0, 27.1; HRMS (ES^+^) *m*/*z*: [M + Na]^+^ calcd for C_41_H_53_O_9_NaSF, 763.3292; found, 763.3310.

**Preparation of (1*S*)-1,5-anhydro-1-{3-[(1-benzothiophen-2-yl)methyl]-4-fluorophenyl}-D-glucitol (6):** A round-bottom flask was charged with compound **5** (20 g, 27 mmol) and methanol (100 mL), then, sodium methoxide (2.92 g, 2.0 equiv) was added and the mixture was heated at 65 °C for 3 h. After cooling down to room temperature, water (300 mL) was added to the resulting mixture, followed by addition of a seed crystal (50 mg) and stirred for 4 h. The crystals were filtered and dried to obtain a white solid 10.4 g. Yield 95.3%; purity (HPLC): 99.78%; mp 155–156.5 °C; [α]_D_^25^ = +24.5° (*c* 1, CH_3_OH); ^1^H NMR (400 MHz, CDCl_3_) δ 7.73 (d, *J* = 7.6 Hz, 1H), 7.65 (d, *J* =7.6 Hz, 1H), 7.45 (dd, *J* =7.2 Hz, 2.0Hz, 1H), 7.39–7.36 (m, 1H), 7.31–7.22 (m, 2H), 7.10 (t, *J* = 9.6 Hz, 1H), 7.05 (s, 1H), 4.31–4.22 (m, 1H), 4.14 (d, *J* = 9.6 Hz, 1H), 3.89 (dd, *J* = 12.4 Hz, 2.0 Hz, 1H), 3.72 (dd, *J* = 12.0 Hz, 5.6 Hz, 1H), 3.51–3.33 (m, 4H); ^13^C NMR (100 MHz, CDCl_3_) δ 161.6, 159.2, 143.6, 140.1, 139.7, 135.8, 130.4, 128.0, 126.1, 123.8, 123.4, 122.6, 121.6, 114.5, 81.5, 80.8, 78.4, 75.1, 70.5, 61.7, 29.3; HRMS (ES^+^) *m*/*z*: [M + Na]^+^ calcd for C_21_H_21_O_5_NaSF, 427.0991; found, 427.0983.

**Preparation of ipragliflozin L-proline (1):** A round-bottom flask was charged with compound **6** (20 g, 49.4 mmol), and filled up with ethanol (100 mL), and then L-proline (5.7 g, 1.0 equiv) and water (10 mL) were added. The mixture was stirred at 100 °C for 0.5 h. Thereafter, the reaction solution was cooled to room temperature, and crystallized to get a white solid 21.5 g. Yield 83.7%; purity (HPLC): 99.92%; decomposition temperature 203.3–205 °C; [α]_D_^25^ = −9.2° (*c* 1, acetonitrile/H_2_O 7:3); ^1^H NMR (400 MHz, CDCl_3_) δ 7.75 (d, *J* = 8.0 Hz, 1H), 7.67 (d, *J* = 7.2 Hz, 1H), 7.45 (d, *J* = 7.6 Hz, 2.4 Hz, 1H), 7.39–7.36 (m, 1H), 7.31–7.23 (m, 2H), 7.10 (t, *J* = 9.2 Hz, 1H), 7.07 (s, 1H), 4.32–4.23 (m, 2H), 4.14 (d, *J* = 9.2 Hz, 1H), 4.00–3.97 (m, 1H), 3.89 (dd, *J* = 12.0 Hz, 1.6 Hz, 1H), 3.71 (dd, *J* = 12.0 Hz, 5.2 Hz, 1H), 3.50–3.31 (m, 5H), 3.27–3.20 (m, 1H), 2.35–2.26 (m, 1H), 2.17–2.09 (m, 1H), 2.01–1.94 (m, 2H); ^13^C NMR (100 MHz, CDCl_3_) δ 172.6, 161.6, 159.2, 143.6, 139.9, 135.9, 130.4, 127.9, 126.0, 123.8, 123.4, 122.6, 121.5, 114.5, 81.5, 80.8, 78.4, 75.1, 70.5, 61.5, 45.6, 29.2, 23.7; MS (ES^+^) *m*/*z*: 427.16 [M + Na]^+^.

**Preparation of (1*R*)-1,5-anhydro-1-{3-[(1-benzothiophen-2-yl)methyl]-4-fluorophenyl}-3,4,6-tri-O-pivaloyl-D-glucitol (8):** A dry round-bottom flask was charged with solid ZnBr_2_ (2.59 g, 1.15 equiv), LiBr (1 g, 1.15 equiv) and anhydrous *n*-dibutyl ether (15 mL) under nitrogen atmosphere, the resulting mixture was stirred at 50 °C for 2 h. After cooling to room temperature, the mixture was used as such for the next step. A solution of compound **4a** (4.05 g, 1.1 equiv) dissolved in anhydrous toluene (20 mL) under nitrogen atmosphere was cooled to 0 °C, *n*-butyllithium in hexane 1.6 M (7.2 mL, 1.15 equiv) was added dropwise over 8 min. After beeing stirred for 2 h, a solution of ZnBr_2_–LiBr in *n*-dibutyl ether was added dropwise over 10 min at 0 °C. After being stirred for 1.5 h, a solution of compound **7** (4.15 g, 10 mmol) in toluene (10 mL) was added to the reaction mixture. The mixture was stirred at room temperature for about 14 h, then saturated ammonium chloride (50 mL) was added (quenched the reaction), the organic phase was washed with water, dried over Na_2_SO_4_ and concentrated under reduced pressure. The residue was purified by column chromatography to afford the title compound as light yellow oil 1.316 g. Yield 20%; purity (HPLC): 98.2%; [α]_D_^25^ = +21.5° (*c* 1, CHCl_3_); ^1^H NMR (400 MHz, CDCl_3_) δ 7.72 (d, *J* = 8.0 Hz, 1H), 7.64 (d, *J* = 7.2 Hz, 1H), 7.46–7.39 (m, 2H), 7.30–7.23 (m, 2H), 7.07 (t, *J* = 9.2 Hz, 1H), 7.01 (s, 1H), 5.24 (t, *J* = 6.0 Hz, 1H), 5.06 (d, *J* = 2.8 Hz, 1H), 4.96 (t, *J* = 4.8 Hz, 1H), 4.57 (m, 1H), 4.29–4.20 (m, 2H), 4.05–3.97 (m, 3H), 2.51 (brs, 1H), 1.24 (s, 9H), 1.19 (s, 9H), 1.14 (s, 9H); ^13^C NMR (100 MHz, CDCl_3_) δ 178.1, 177.4, 176.6, 161.5, 159.1, 143.3, 140.0, 139.7, 133.0, 130.1, 127.7, 126.6, 123.9, 123.0, 122.1, 121.8, 115.5, 72.8, 71.9, 71.0, 69.9, 67.2, 60.7, 38.8, 30.2, 27.1; MS (ES^−^) *m*/*z*: 691.09 [M + Cl]^−^.

**Preparation of (1*R*)-1,5-anhydro-1-{3-[(1-benzothiophen-2-yl)methyl]-4-fluorophenyl}-D-glucitol (6’):** A round-bottom flask was charged with compound **8** (1.316 g, 2 mmol) and filled up with methanol (7 mL), and then sodium methoxide (0.324 g, 3.0 equiv) was added, the resulting mixture was heated at 65 °C for 3 h. After cooling down to room temperature, the reaction was quenched with a solution of ammonium chloride (10 wt %, 15 mL). The aqueous layer was extracted with ethyl acetate (15 mL). The organic layer was washed with water, dried over Na_2_SO_4_ and concentrated under reduced pressure. The residue was purified by column chromatography to afford the title compound as light yellow solid 0.602 g. Yield 74.4%; purity (HPLC): 99.8%; mp 164.5–167.6 °C; [α]_D_^25^ = +55.5° (*c* 1, MeOH); ^1^H NMR (400 MHz, CD_3_OD) δ 7.74 (d, *J* = 7.6 Hz, 2H), 7.70–7.66 (m, 2H), 7.31–7.22 (m, 2H), 7.10–7.06 (m, 2H), 5.08 (d, *J* = 4.0 Hz, 1H), 4.26 (s, 2H), 3.90–3.83 (m, 2H), 3.79–3.69 (m, 2H), 3.48–3.42 (m, 2H); ^13^C NMR (100 MHz, CD_3_OD) δ 161.1, 158.6, 143.7, 140.1, 139.7, 134.8, 130.9, 128.6, 126.0, 123.8, 123.4, 122.6, 121.5, 114.4, 75.7, 73.7, 72.9, 61.1, 29.4; MS (ES^+^) *m*/*z*: 427.33 [M + Na]^+^.

**Preparation of (1*R*)-1,5-anhydro-1-{3-[(1-benzothiophen-2-yl)methyl]-4-fluorophenyl}-2,3,4,6-tetra-*O*-pivaloyl-D-glucitol (5’):** In a round-bottom flask, a mixture of compound **8** (1 g, 1.52 mmol), DCM (5 mL), DMAP (10 mg, 0.05 equiv) and TEA (0.46 g, 3.0 equiv) were stirred at room temperature. Then, pivaloyl chloride (0.55 g, 3.0 equiv) was added dropwise and the mixture was stirred at room temperature for 5 days, the reaction was diluted with DCM (10 mL) and quenched with saturated ammonium chloride (10 mL), the organic layer was washed with water, dried over Na_2_SO_4_ and concentrated under reduced pressure. The residue was purified by column chromatography to afford the title compound as light yellow solid 0.596 g. Yield 52.84%; purity (HPLC): 95.7%; mp 159.2–162.3 °C; [α]_D_^25^ = +78.9° (*c* 1, CHCl_3_); ^1^H NMR (400 MHz, CDCl_3_) δ 7.72 (d, *J* = 8 Hz, 1H), 7.65 (d, *J* = 7.6 Hz, 1H), 7.59 (dd, *J* = 6.8 Hz, 1.6 Hz, 1H), 7.52–7.48 (m, 1H), 7.30–7.22 (m, 2H), 7.12–7.07 (m, 2H), 5.69 (t, *J* = 9.2 Hz, 1H), 5.35 (dd, *J* = 9.2 Hz, 5.6 Hz, 1H), 5.29 (d, *J* = 6.0 Hz, 1H), 5.16 (t, *J* = 9.2 Hz, 1H), 4.31–4.23 (m, 2H), 4.11–4.02 (m, 2H), 3.64–3.60 (m, 1H), 1.18 (s, 9H), 1.17 (s, 9H), 1.13 (s, 9H), 0.92 (s, 9H); ^13^C NMR (100 MHz, CDCl_3_) δ 178.0, 177.1, 176.6, 161.6, 159.1, 142.8, 139.9, 131.4, 129.0, 126.9, 124.0, 123.1, 122.1, 115.7, 73.1, 70.6, 68.4, 62.1, 38.7, 30.0, 27.0; MS (ES^+^) *m*/*z*: 763.44 [M + Na]^+^.

## Supporting Information

File 1 ^1^H NMR, ^13^C NMR and HRMS spectra of compounds **1**, **5**, **6**, **5’**, **6’** and **8**, and HPLC diagram of **5**.
