# Supplementary material for: A concise and practical stereoselective synthesis of ipragliflozin L-proline
Source: Beilstein J Org Chem. 2017 Jun 1;13:1064–70. doi: 10.3762/bjoc.13.105 (PMC5480354; doi:10.3762/bjoc.13.105)
Supplement: File 1 — 1H NMR, 13C NMR and HRMS spectra of compounds 1, 5, 6, 5’, 6’ and 8, and HPLC diagram of 5. [file Beilstein_J_Org_Chem-13-1064-s001.pdf]

Supporting Information

for

A concise and practical stereoselective synthesis of ipragliflozin L-proline

Shuai Ma, Zhenren Liu, Jing Pan, Shunli, Zhang, Weicheng Zhou*

Address: State Key Lab of New Drug & Pharmaceutical Process, Shanghai Key Lab of Anti-Infectives, Shanghai Institute of Pharmaceutical Industry, China State Institute of Pharmaceutical Industry, No. 285, Gebaini Rd., Shanghai 201203, P. R. of China

Email: Weicheng Zhou*- zhouweicheng58@163.com

*Corresponding author

^{1}H NMR, ^{13}C NMR and HRMS spectra of compounds 1, 5, 6, 5', 6' and 8, and HPLC diagram of 5

Table of contents

^{1}H NMR and ^{13}C NMR spectrum of compound **5** S3

HRMS spectrum of compound **5** S4

HPLC spectrum of compound **5** S5

^{1}H NMR and ^{13}C NMR spectrum of compound **6** S6

HRMS spectrum of compound **6** S7

^{1}H NMR and ^{13}C NMR spectrum of compound **1** S8

^{1}H NMR and ^{13}C NMR spectrum of compound **8** S9

^{1}H NMR and ^{13}C NMR spectrum of compound **6’** S10

^{1}H NMR and ^{13}C NMR spectrum of compound **5’** S11

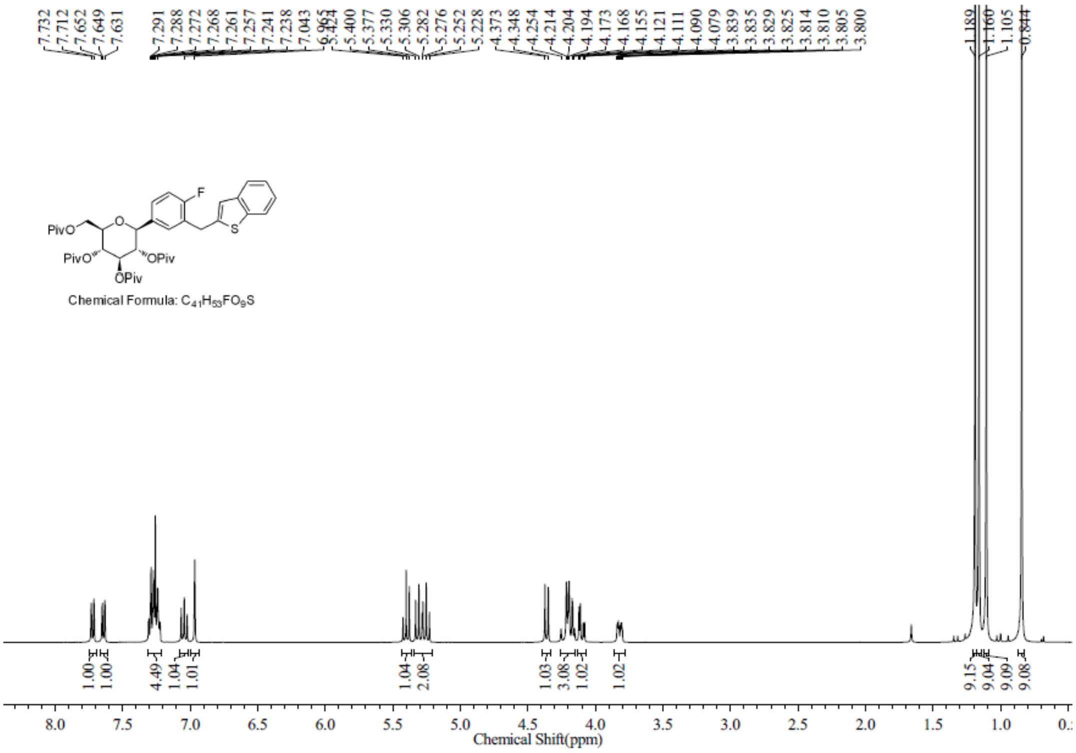

^{1}H NMR spectrum of compound **5**

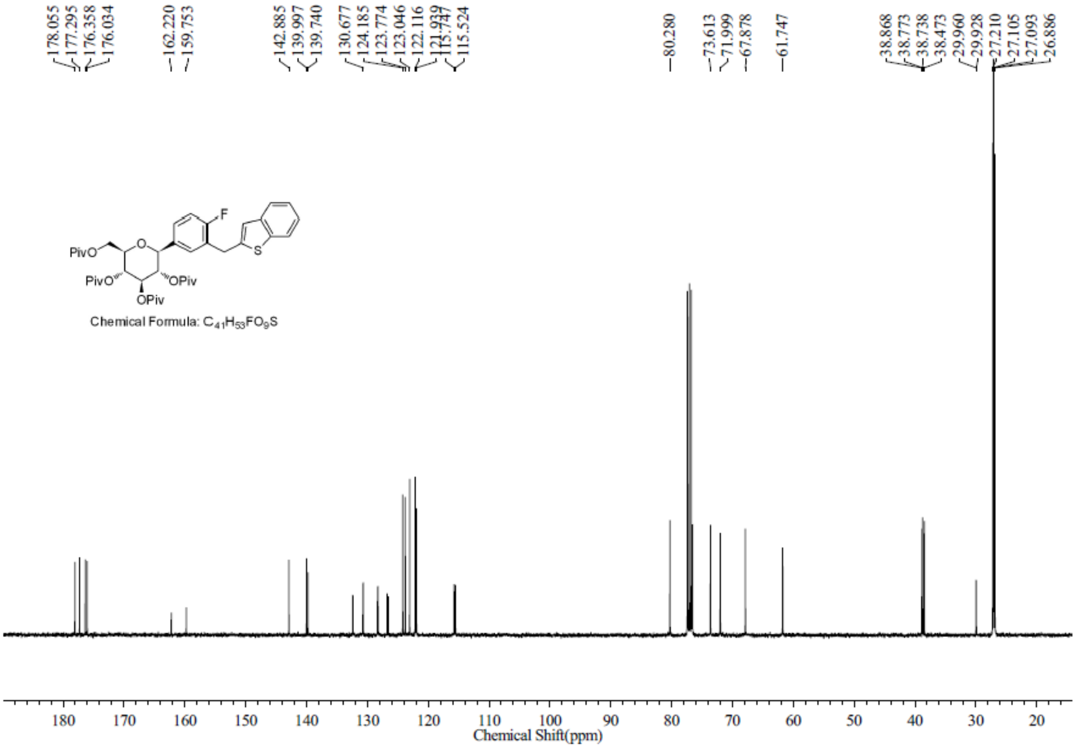

^{13}C NMR spectrum of compound **5**

Elemental Composition Report

Single Mass Analysis
Tolerance = 5.0 PPM / DBE: min = -1.5, max = 50.0
Element prediction: Off
Number of isotope peaks used for i-FIT = 3

Monoisotopic Mass, Even Electron Ions
170 formula(e) evaluated with 2 results within limits (up to 50 closest results for each mass)
Elements Used:
C: 5-55 H: 5-65 O: 1-9 Na: 1-1 S: 0-1 F: 0-1

SIPI
I5
Q16-1771HR 14 (0.260) AM (Cen,4, 80.00, Ar,5000.0,767.35,1.00); Sm (Mn, 2x3.00); Sb (1,40.00); Cm (4:25)

Q-Tof micro
YA019

08:25:33,23-Nov-2016
TOF MS ES+
4.00e+004

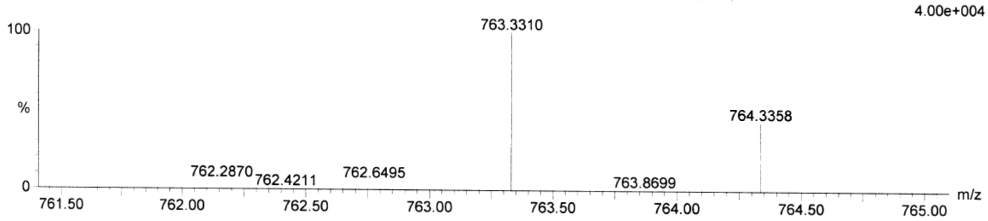

Minimum:				-1.5		
Maximum:		5.0	5.0	50.0		
Mass	Calc. Mass	mDa	PPM	DBE	i-FIT	Formula
763.3310	763.3292	1.8	2.4	14.5	n/a	C41 H53 O9 Na S F

HRMS spectrum of compound **5**

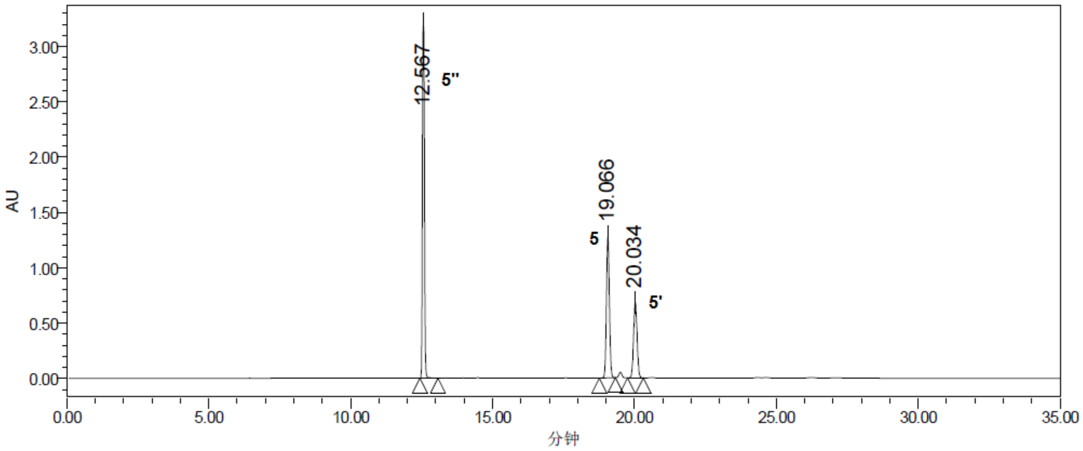

峰结果

	保留时间 (分钟)	名称	面积 (微伏*秒)	高度 (微伏)	% 面积
1	12.567		16130039	3205166	51.47
2	19.066		9336482	1277501	29.79
3	20.034		5873495	681279	18.74

HPLC spectrum of compounds **5, 5’** and **5”**

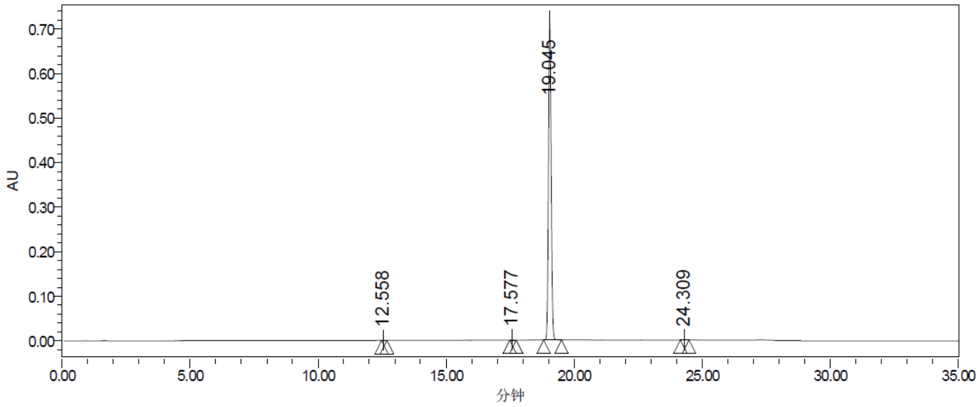

峰结果

	保留时间 (分钟)	面积 (微伏*秒)	高度 (微伏)	% 面积
1	12.558	2239	558	0.04
2	17.577	3983	744	0.08
3	19.045	5247427	715860	99.79
4	24.309	4739	454	0.09

HPLC spectrum of compound **5**

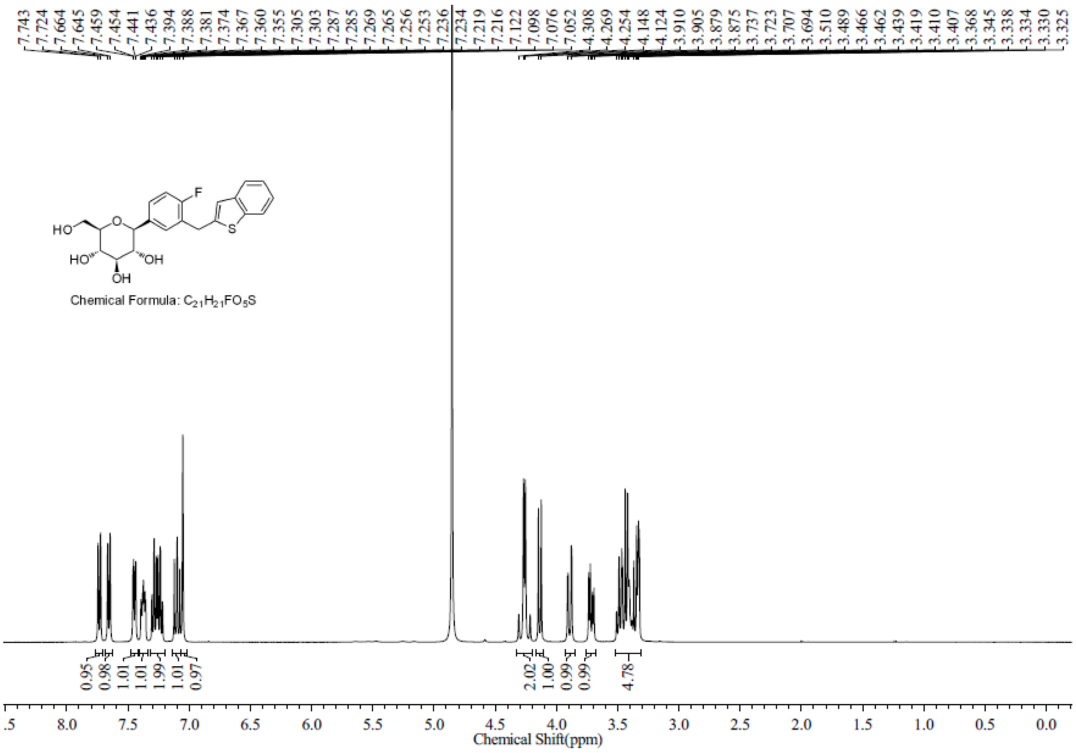

^{1}H NMR spectrum of compound **6**

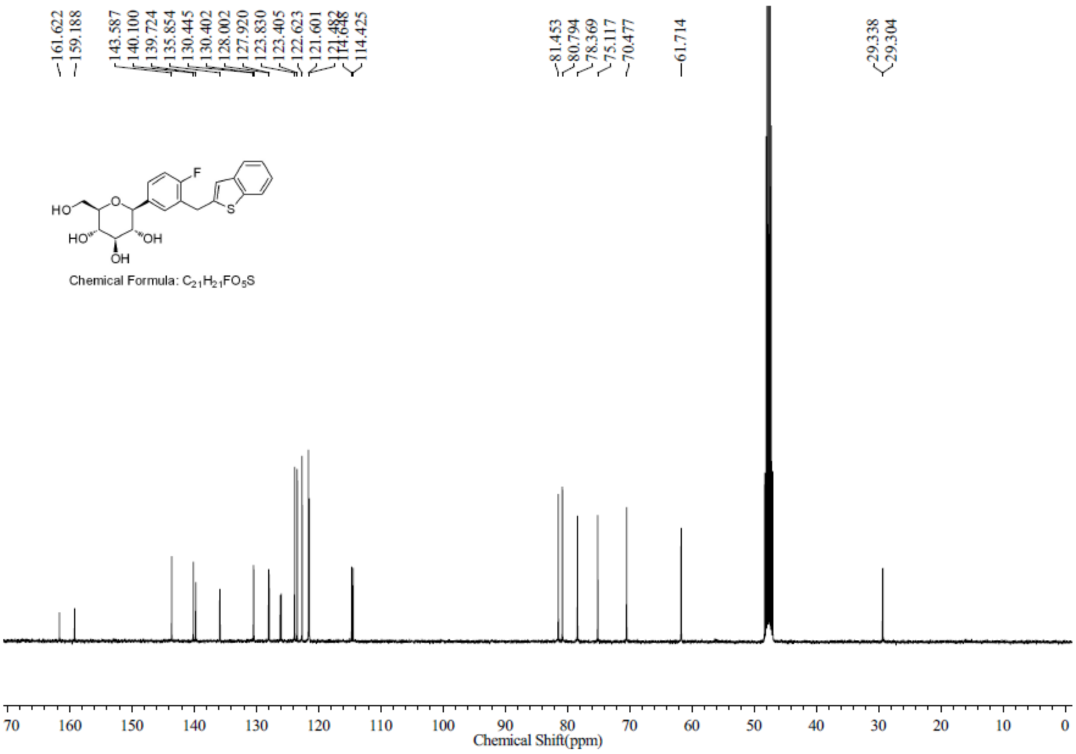

^{13}C NMR spectrum of compound **6**

Elemental Composition Report Page 1

Single Mass Analysis
Tolerance = 5.0 PPM / DBE: min = -1.5, max = 50.0
Element prediction: Off
Number of isotope peaks used for i-FIT = 3

Monoisotopic Mass, Even Electron Ions
50 formula(e) evaluated with 2 results within limits (up to 50 closest results for each mass)
Elements Used:
C: 5-35 H: 5-35 O: 1-5 F: 0-1 Na: 1-1 S: 0-1

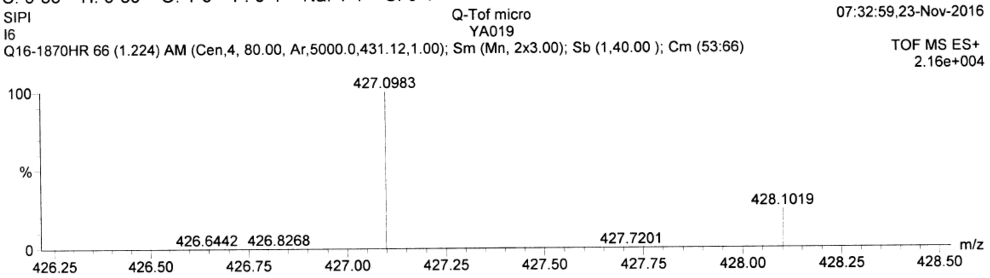

Mass	Calc. Mass	mDa	PPM	DBE	i-FIT	Formula
Minimum:				-1.5		
Maximum:		5.0	5.0	50.0		
427.0983	427.0980	0.3	0.7	14.5	n/a	C24 H20 O4 Na S
	427.0991	-0.8	-1.9	10.5	n/a	C21 H21 O5 F Na S

HRMS spectrum of compound **6**

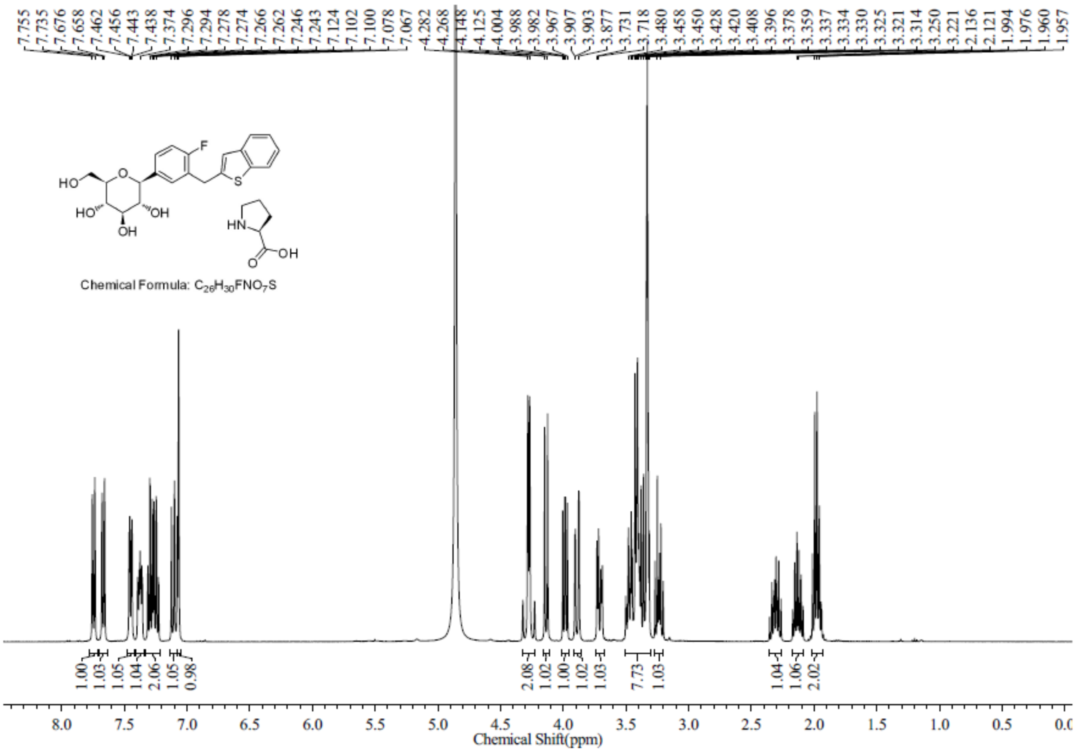

^{1}H NMR spectrum of compound **1**

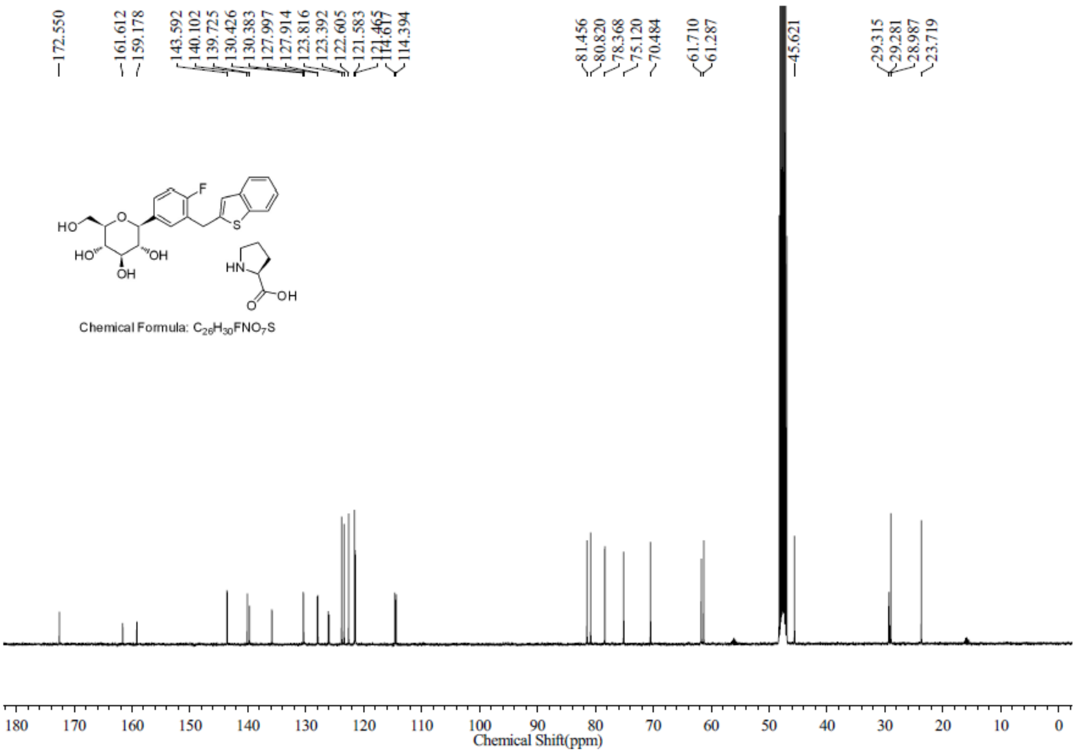

^{13}C NMR spectrum of compound **1**

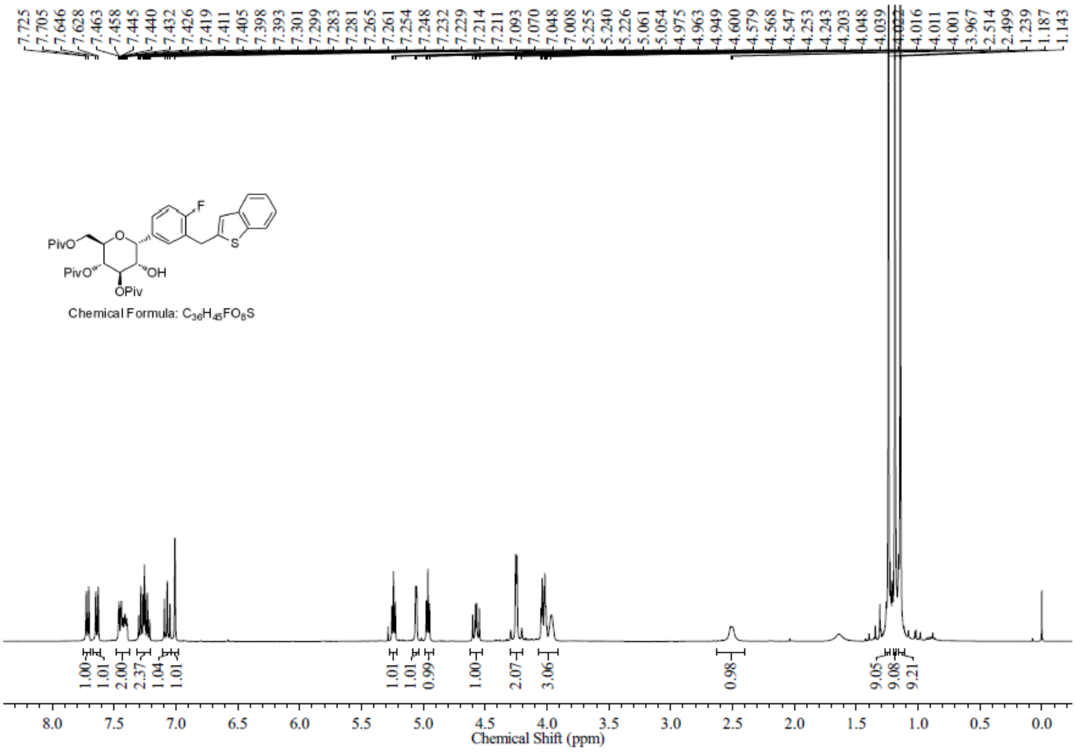

^{1}H NMR spectrum of compound **8**

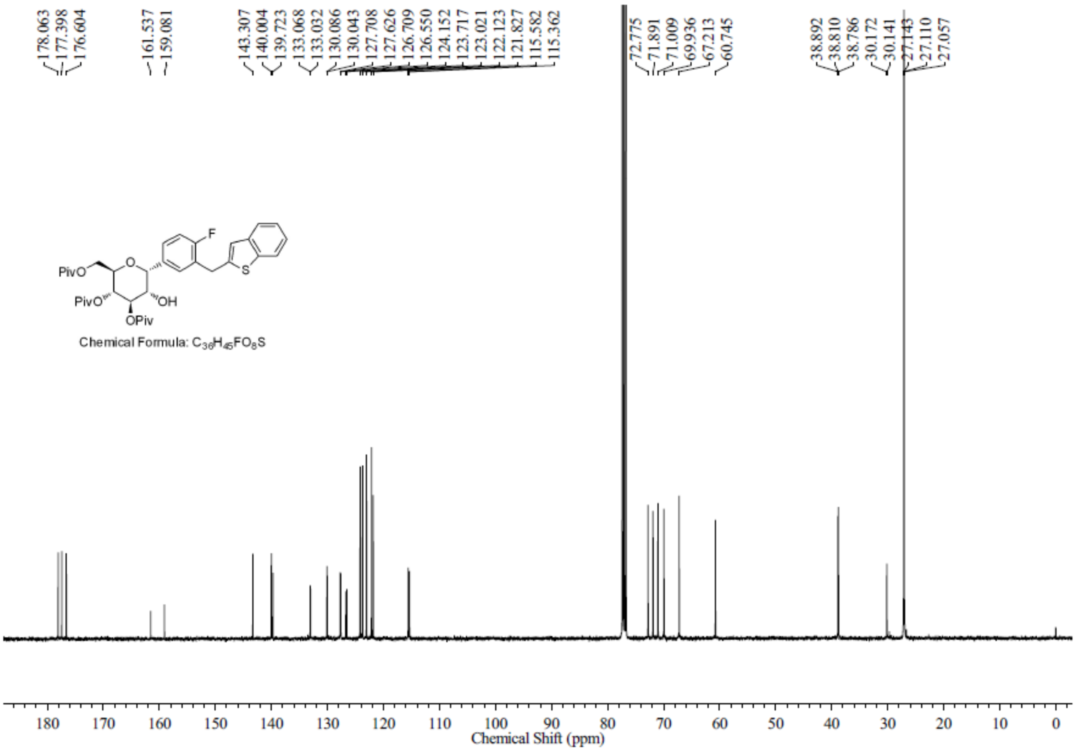

^{13}C NMR spectrum of compound **8**

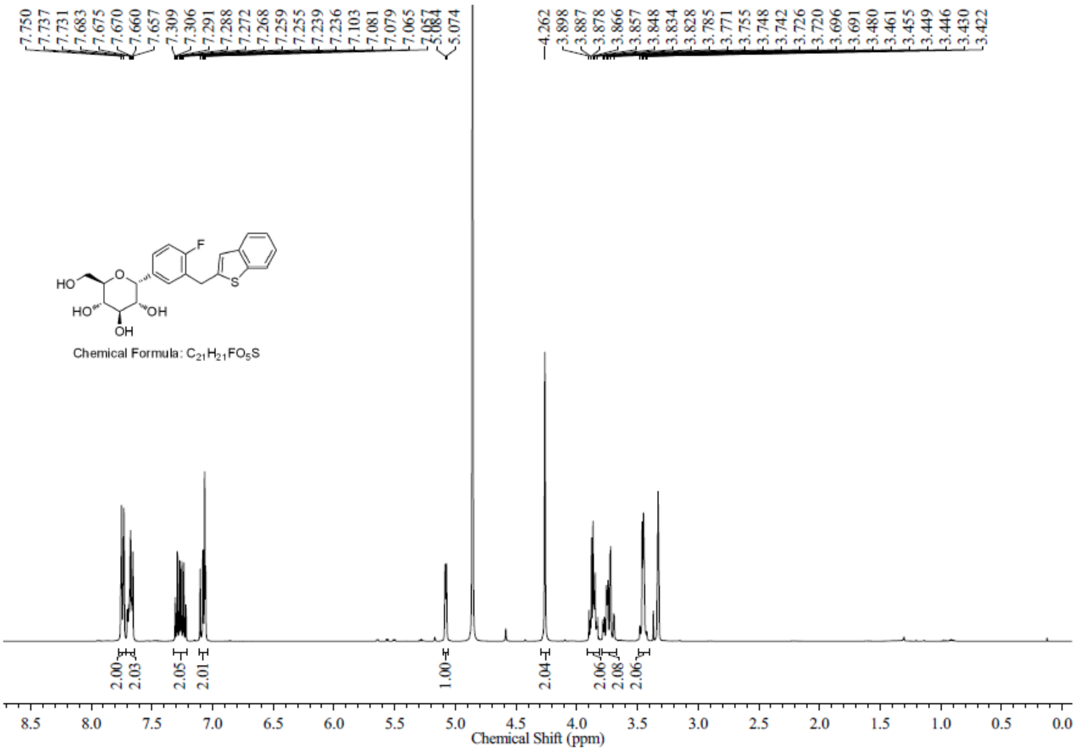

^{1}H NMR spectrum of compound **6’**

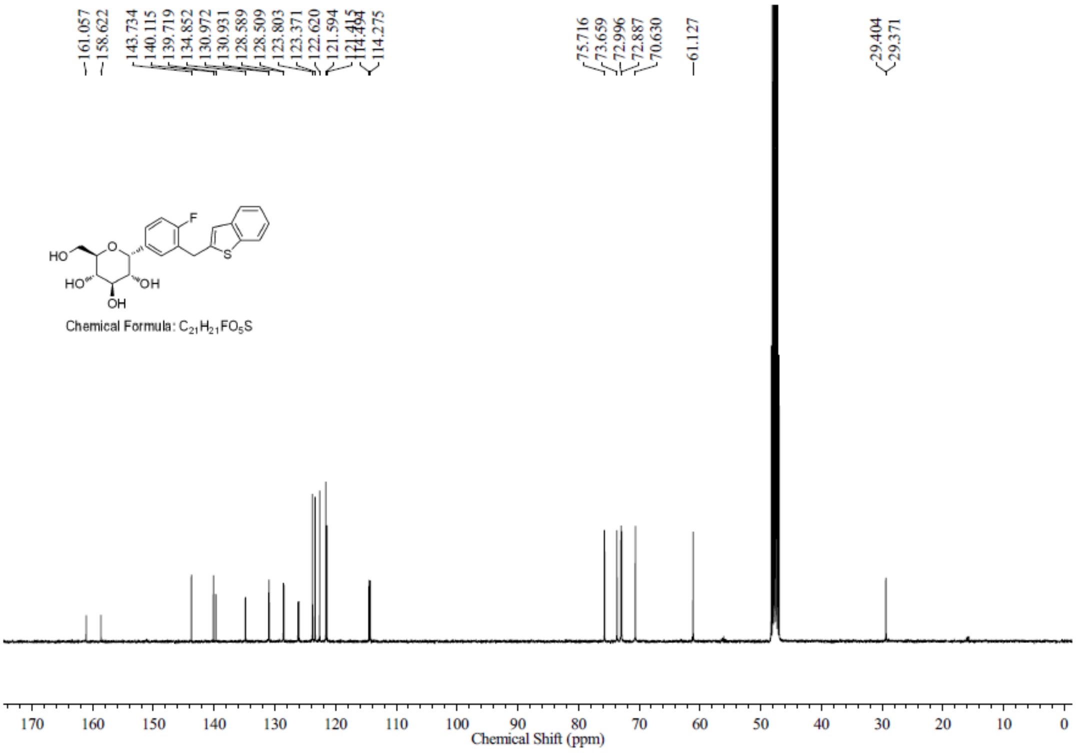

^{13}C NMR spectrum of compound **6’**

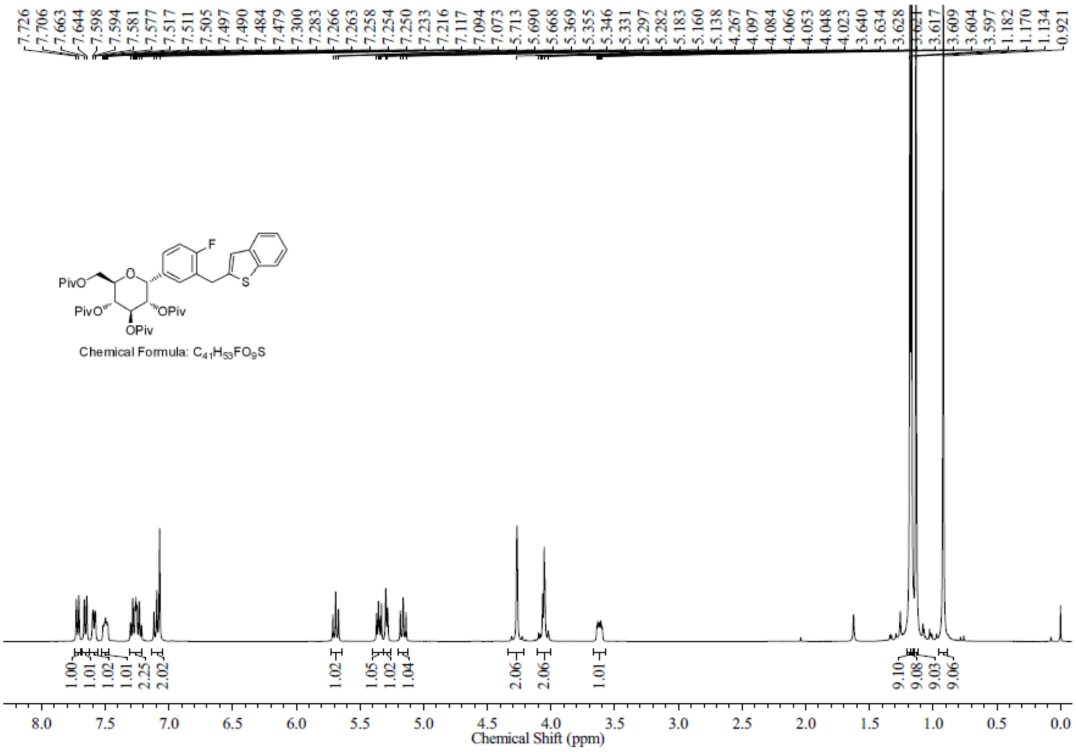

^{1}H NMR spectrum of compound **5’**

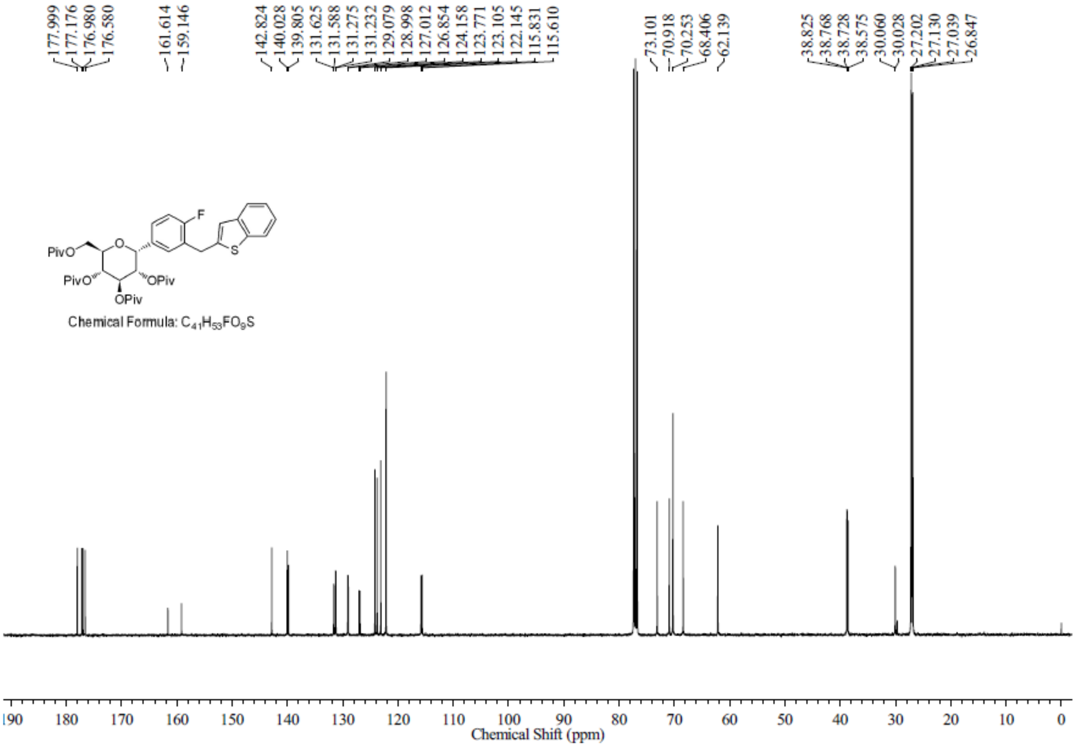

^{13}C NMR spectrum of compound **5’**
